# Cryo-EM structures of cardiac muscle α-actin mutants M305L and A331P give insights into the structural mechanisms of hypertrophic cardiomyopathy

**DOI:** 10.1016/j.ejcb.2024.151460

**Published:** 2024-12

**Authors:** Hsiang-Ling Huang, Andrejus Suchenko, Giovanna Grandinetti, Mohan K. Balasubramanian, Krishna Chinthalapudi, Sarah M. Heissler

**Affiliations:** a Department of Physiology and Cell Biology, Dorothy M. Davis Heart and Lung Research Institute, College of Medicine, The Ohio State University, Columbus, OH, USA; b Centre for Mechanochemical Cell Biology and Warwick Medical School, Division of Biomedical Sciences, Coventry, United Kingdom; c Center for Electron Microscopy and Analysis, College of Engineering, The Ohio State University, Columbus, OH, USA

**Keywords:** Actin, Cytoskeleton, Mutation, Myosin, Hypertrophic cardiomyopathy

## Abstract

Cardiac muscle α-actin is a key protein of the thin filament in the muscle sarcomere that, together with myosin thick filaments, produce force and contraction important for normal heart function. Missense mutations in cardiac muscle α-actin can cause hypertrophic cardiomyopathy, a complex disorder of the heart characterized by hypercontractility at the molecular scale that leads to diverse clinical phenotypes. While the clinical aspects of hypertrophic cardiomyopathy have been extensively studied, the molecular mechanisms of missense mutations in cardiac muscle α-actin that cause the disease remain largely elusive. Here we used cryo-electron microscopy to reveal the structures of hypertrophic cardiomyopathy-associated actin mutations M305L and A331P in the filamentous state. We show that the mutations have subtle impacts on the overall architecture of the actin filament with mutation-specific changes in the nucleotide binding cleft active site, interprotomer interfaces, and local changes around the mutation site. This suggests that structural changes induced by M305L and A331P have implications for the positioning of the thin filament protein tropomyosin and the interaction with myosin motors. Overall, this study supports a structural model whereby altered interactions between thick and thin filament proteins contribute to disease mechanisms in hypertrophic cardiomyopathy.

## Highlights

• M305L and A331P have different impacts on the architecture of the actin filament.
• Mutation-specific allosteric changes are communicated to protomer interfaces.
• M305L and A331P have distinct interaction mechanisms with myosin and tropomyosin.

## Graphical Abstract

**Figure:**
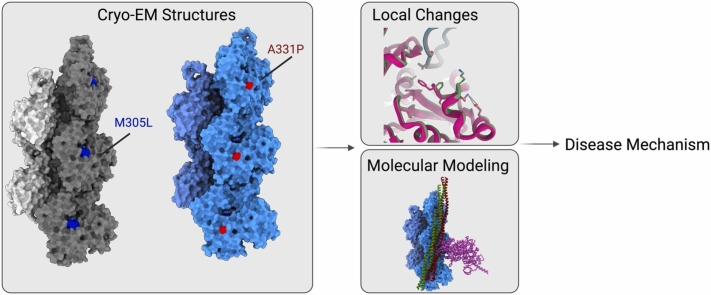

## 1 Introduction

Cardiac muscle α-actin is essential for the normal structure and function of the heart muscle (Sequeira et al., 2014). It accounts for ∼80 % of the total actin in healthy and diseased hearts where it is almost exclusively found in the thin filaments of the sarcomere, the basic contractile apparatus of cardiomyocytes (Alonso et al., 1990, Vandekerckhove et al., 1986). Thin filaments are primarily composed of cardiac muscle α-actin and the regulatory proteins tropomyosin and troponin. Thin filaments together with myosin thick filaments are the major components of the muscle sarcomere. The relative sliding of thin and thick filaments past one another generates force and muscle contraction important for normal heart function (Gordon et al., 2000, Huxley and Niedergerke, 1954, Huxley, 1969). In muscle, the interaction between thick and thin filaments is tightly regulated by the troponin/tropomyosin complex that restricts the binding of myosin to filamentous actin (F-actin) in the blocked state (B-state) under Ca^2+^-free conditions. The presence of Ca^2+^ results in the binding of Ca^2+^ to troponin and enables an ∼25° azimuthal movement of tropomyosin into the closed state (C-state) that partially covers the myosin binding site on F-actin. Myosin binding is associated with an additional ∼10° azimuthal movement of tropomyosin into the energetically unfavorable open state (M-state) that results in the actin-activation of the myosin ATPase activity and thus force production and muscle contraction (Li et al., 2011, McKillop and Geeves, 1993, von der Ecken et al., 2016, von der Ecken et al., 2015, Yamada et al., 2020).

Mutations in thin and thick filament proteins have been shown to cause a diverse range of diseases of the heart, including hypertrophic cardiomyopathy (HCM) (Mogensen et al., 1999, Olson et al., 2000). HCM affects approximately 1 in 500 individuals worldwide and is the most common form of genetic heart disease (Semsarian et al., 2015). HCM has been linked to hypercontractility at the molecular scale. While the effect of HCM mutations in myosin thick filaments has been extensively studied, our knowledge of HCM mutations in thin filaments is limited (Lehman et al., 2023, Lowey et al., 2018, Morck et al., 2022, Spudich, 2015, Trivedi et al., 2018). Mutations in thin filament proteins have been shown to cause ∼20 % of cases of HCM out of which up to 5 % of HCM cases have been attributed to mutations in the cardiac muscle α-actin gene *ACTC1* (Van Driest et al., 2003). These include the clinically relevant missense mutations M305L and A331P (Despond and Dawson, 2018, Mogensen et al., 1999, Olson et al., 2000). Given the high conservation of the actin molecule and its key function in sarcomere contraction, it is conceivable that missense mutations in cardiac muscle α-actin modulate the interactions with regulatory thin filament proteins and/or myosin. Indeed, several studies reported biochemical alterations in the interaction between cardiac muscle α-actin mutants and contractile proteins of the sarcomere (Bookwalter and Trybus, 2006, Chow et al., 2014, Dahari and Dawson, 2015, Debold et al., 2010, Despond and Dawson, 2018, Greve et al., 2024b, Liu et al., 2018, Muller et al., 2012, Mundia et al., 2012). However, the structural mechanisms by which both mutations dysregulate heart function in HCM at the molecular scale remain largely unknown.

To gain a comprehensive structural understanding of the effect of HCM-causing mutations M305L and A331P in cardiac muscle α-actin, we used cryo-electron microscopy (cryo-EM) to solve their structures. The comparison of our 3.12 Å (M305L) and 3.42 Å (A331P) structures with our recent 3.0 Å resolution structure of native cardiac muscle α-actin (PDB ID: 8DMY) reveals subtle changes that locally modulate the structure throughout the protomer and the filament. Structural superimposition reveals mutation-specific interaction profiles with the myosin motor domain and the positioning of tropomyosin in different functional states. Thus, our work broadens our understanding of the effect of mutations on actin structure and function in the context of HCM with potential relevance to other cardiac disorders and nonmuscle actinopathies.

## 2 Material and methods

### 2.1 Cloning and site-directed mutagenesis

The human cardiac muscle α-actin (*ACTC1*) coding sequence was codon optimized for the expression in mammalian cells. The *ACTC1* coding sequence was synthesized as gBlock (IDT) fused to a linker sequence, thymosin β4 and His tag at the C-terminus and cloned into pcDNA3 plasmid. The design of the expression construct allows for the seamless removal of the linker sequence, the thymosin β4 and His tag from cardiac muscle α-actin due to the presence of a naturally occurring chymotrypsin cleavage site at the very C-terminus of the actin coding sequence. Missense mutations M305L and A331P were introduced in the corresponding codons in the cardiac muscle α-actin cDNA with site-directed mutagenesis. Fully complementary primers with a respective mutation (IDT) were used to amplify the pcDNA3-ACTC1-Tβ4-His plasmid with Phusion high fidelity DNA polymerase (NEB). All expression plasmids were sequence verified prior to protein production.

### 2.2 Protein production and purification

Proteins were recombinantly produced in human embryonic kidney (HEK) cells that have the necessary enzymes to properly fold and post-translationally process actin isoforms (Ceron et al., 2022). In brief, plasmid DNA was transfected into HEK293T cells at a confluency of 90 %. DNA plasmid was mixed with polyethyleneimine (PEI) at a ratio of 1:3 (μg DNA:μg PEI) incubated for 15 minutes at room temperature and added into a T175 flask with cells. Transfected cells were incubated for 72 hours before harvesting. For protein purification, HEK293T cells producing human cardiac muscle α-actin were collected using TrypLE cell dissociation agent (Gibco). The cells were pelleted and washed twice with ice-cold PBS. Washed cells were lysed in 3 ml ice-cold lysis buffer (150 mM NaCl, 1 % Triton X-100, 50 mM Tris-HCl pH 7.8) with 1x protease inhibitor cocktail (Roche). The cell lysate was centrifuged at 15k rpm at 4 °C for 1 h. Then the supernatant was mixed with 2x binding buffer (20 mM imidazole, 20 mM HEPES pH 7.4, 600 mM NaCl, 4 mM MgCl_2_, 2 mM ATP, 2x concentration of protease inhibitor cocktail (Roche) 1 mM phenylmethylsulfonyl fluoride (PMSF), 7 mM β-mercaptoethanol (β-ME)) and filtered through a 0.22 μm syringe filter. The filtrate was incubated with 0.5 ml of nickel resin (Thermo Fisher Scientific) for 1 h on a roller at 4 C. The nickel resin was pelleted and washed twice with 1x binding buffer (without protease inhibitor cocktail and PMSF) and then three times with ice-cold G buffer (5 mM HEPES pH 7.4, 0.2 mM CaCl_2_, 0.01 % (w/v) NaN_3_, 0.2 mM ATP, 0.5 mM dithiothreitol (DTT)). The sample was resuspended in 10 ml of G buffer and incubated overnight with chymotrypsin (Sigma). The chymotrypsin was inactivated with PMSF and the sample was concentrated with a 30 kDa cut Amicon Ultra 15 filter unit (Merck) to a volume of 1 ml. The purified protein was polymerized by the addition of 10x MEK buffer (20 mM MgCl_2_, 50 mM glycol-bis(2- aminoethylether)-N,N,N′,N′-tetraacetic (EGTA), 1 M KCl) for 1 h at room temperature. Then the filamentous actin was pelleted at 100,000 g at 4 °C for 1 h. The pellet was collected and depolymerized by dialysis for two days in G buffer. Monomeric actin was aliquoted and snap-frozen in liquid nitrogen and stored at −80 °C. A yield of 0.5 mg of recombinant cardiac muscle α-actin was obtained from 2.5·10^8^ cells.

### 2.3 Cryo-EM grid preparation and data collection

For cryo-EM data collection, the G-actin of actin mutants M305L and A331P was polymerized with 10x KMEM buffer containing 100 mM MOPS pH 7.2, 20 mM MgCl_2_, 50 mM EDTA, and 1 M KCl. C-flat grids (Au, 300-mesh, CF-1.2/1.3, Electron Microscopy Sciences) were glow-discharged with a PELCO easiGlow (TedPella) at 20 mA and 0.39 mBar for 1 min. 4 µL of protein samples were applied to freshly glow-discharged grids, incubated for 1 min, and blotted for 5 s at 99 % humidity at 18˚C. Grids were plunge-frozen in liquid ethane with a Leica EM GM2 plunger (Leica Microsystems) and stored in liquid nitrogen until data collection. All grids were screened on a 200 kV Glacios cryo-TEM (Thermo Fisher Scientific) at a magnification of 92,000× with a Falcon 3EC detector. Data were collected on optimal grids on a 300 kV Krios G3i (Thermo Fisher Scientific) equipped with a K3 detector (Gatan) using a magnification of 81,000x in super-resolution mode with a pixel size of 0.4455 Å and a total dose of 60 e^−^/Å^2^ per movie at a defocus range of –0.5 µm to –2.5 µm. EPU software (Thermo Fisher Scientific) was used for data collection. A total of 2898 movies for the A331P mutant and 5275 movies for the M305L mutant were collected.

### 2.4 Cryo-EM data processing and model building

Cryo-EM data processing was performed as described before using CryoSPARC v4 (Arora et al., 2023, Punjani et al., 2017). In brief, all movies were motion corrected using the cryoSPARC patch motion correction and CTF was estimated using the patch CTF estimation routines implemented in CryoSPARC v4 (Punjani et al., 2017). Micrographs with resolutions below 5 Å were discarded using a manual curation routine. Template-free filament tracing was performed using a filament tracer in CryoSPARC. All particles were extracted with a Fourier cropped box size of 64 px and several rounds of 2D classification were performed to remove junk particles or filament segments. *Ab-initio* routines were used to generate the initial model for F-actin and then heterogeneous refinement was used to sort the heterogeneity in the particle population. The best filament class was selected, and homogeneous refinements were performed. 3D classification was performed to sort further heterogeneity in the filament population. For the A331P mutant, 247,719 well-aligned particle stacks were obtained and reextracted with a box size of 256 px. Helical refinement on these particles resulted in a global resolution of 3.42 Å at FSC=0.143. For the M305L mutant, a final stack of 383,990 particles was obtained. Helical refinement on these particles resulted in a global resolution of 3.12 Å at FSC=0.143. Using these final maps, model building was performed using our native cardiac muscle α-actin structure (PDB ID: 8DMY) as a template in Phenix using real-space refinement (Afonine et al., 2018). Residues A331 and M305 were mutated to P331 and L305 in COOT, respectively (Emsley et al., 2010). All models were built iteratively using Phenix and Coot. The final models were validated using MolProbity (Williams et al., 2018). Model building statistics are shown in Table 1.

**Table 1:** Data collection and refinement statistics.

	M305L (EMD-44777) (PDB-9BPH)			A331P (EMD-44781) (PDB-9BPM)
**Data collection and processing**				
Magnification	81,000			81,000
Voltage (kV)	300			300
Electron exposure (e–/Å^2^)	60			60
Defocus range (μm)	–0.5 to –2.5			–0.5 to –2.5
Pixel size (Å)	0.891			0.891
Symmetry imposed	C1			C1
Initial particle images (no.)	761,645			413,256
Final particle images (no.)	383,990			247,719
Map resolution (Å) FSC threshold	3.12 0.143			3.42 0.143
Map resolution range (Å)	1.5–4.0			2.0–4.0
**Refinement**				
Initial model used (PDB code)	8DMY			8DMY
Model resolution (Å) FSC threshold	3.12 0.143			3.42 0.143
Map sharpening *B* factor (Å^2^)	−75			−98
Model composition				
Chains Non-hydrogen atoms Protein residues Ligands	4 11628 1484 8			4 11628 1484 8
*B* factors (Å^2^)				
Protein Ligand	76.2 58.6			77.8 60.1
R.m.s. deviations				
Bond lengths (Å) Bond angles (°)	0.004 0.659			0.003 0.669
Validation				
MolProbity score Clashscore Poor rotamers (%)	1.95 5.23 3.50			1.78 5.87 0.00
Ramachandran plot				
Favored (%) Allowed (%) Disallowed (%)	96.17 3.83 0.00			95.90 4.10 0.00

### 2.5 Normal mode analysis

Normal mode analysis was performed to analyze the motions in actin filaments using DynOmics (Li et al., 2017). Specifically, we used the anisotropic network model (ANM) for predicting the structural dynamics and global motion in the actin filaments.

## 3 Results

### 3.1 Recombinant protein production and cryo-EM of cardiac muscle α-actin mutants

**Fig. 1:**
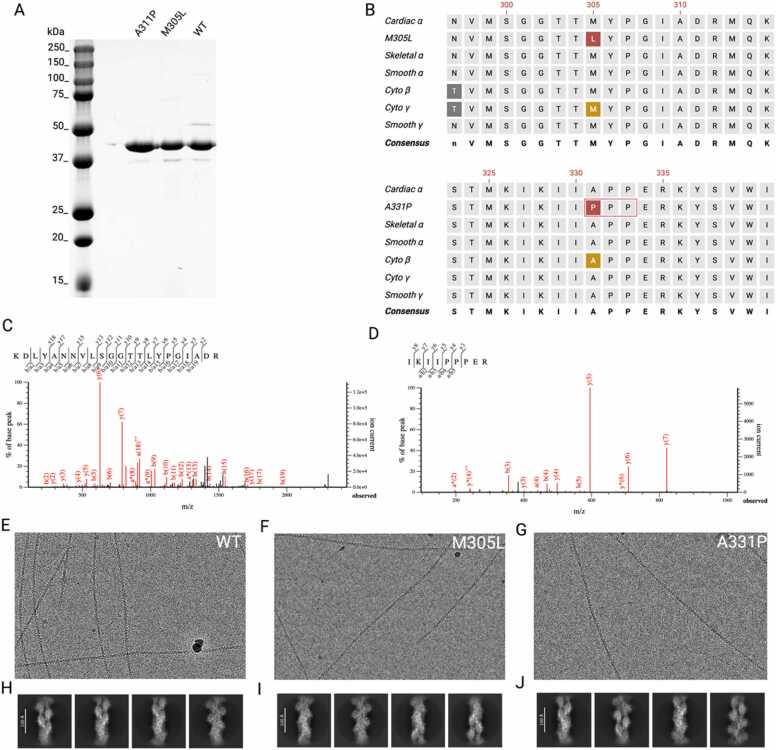
**Protein purification and cryo-EM analysis of cardiac muscle α-actin mutants M305L and A331P. (A)** Representative purifications of native (WT) cardiac muscle α-actin and mutants M305L and A331P resolved on an SDS-PAGE gel. The proteins have an apparent molecular weight of 42 kDa. **(B)** Multiple sequence alignment of human actin isoforms. Numbering according to the sequence of mature cardiac muscle α-actin. Mutations M305L and A331P in the cardiac muscle α-actin sequence are highlighted in red. Sequence deviations from the consensus sequence between actin isoforms are highlighted in dark grey. Reported mutations in residues in homologous positions to cardiac muscle α-actin M305L and A331 are highlighted in orange. The three adjacent proline residues in A331P are indicated with a red box. Cardiac α, cardiac muscle α-actin; M305L, cardiac muscle α-actin harboring mutation M305L; A331P, cardiac muscle α-actin harboring mutation A331P; Skeletal α, skeletal muscle α-actin; Smooth, smooth muscle α-actin; Cyto β, cytoplasmic β-actin; Cyto γ, cytoplasmic γ-actin; Smooth γ, smooth muscle γ-actin. Capital letters in the consensus sequence indicate identical residues, small letters indicate positions with variable residues within native actin isoform sequences. **(C)** The mass spectrum shows the presence of the M305L mutation in recombinant cardiac muscle α-actin. **(D)** The mass spectrum shows the presence of the A331P mutation in recombinant cardiac muscle α-actin. (**E-G**) Representative cryo-EM micrographs of WT, M305L, and A331P. (**H-J**) Representative 2D class averages of WT, M305L, and A331P.

Plasmids for the recombinant production of cardiac muscle α-actin mutants M305L and A331P were generated by site-directed mutagenesis and the respective proteins were recombinantly produced in HEK293T cells. A multi-step purification strategy resulted in homogeneous, tag-free protein purifications of mature globular actin (G-actin) with an apparent molecular weight of 42 kDa as shown by SDS-PAGE (Fig. 1A,B) (Hatano et al., 2020). The identity of the purified cardiac muscle α-actin was confirmed by mass spectrometry (Fig. 1C,D).

Following our established procedures, we subjected M305L and A331P to cryo-EM analysis (Arora et al., 2023). The cryo-EM micrographs showed the ability of the recombinant cardiac muscle α-actin mutants to polymerize into long filaments with the characteristic appearance of F-actin observed in wild-type cardiac muscle α-actin (WT) (Fig. 1E,F,G). Changes in appearance including short, curved, or breaks within the mutant actin filaments were not observed in comparison to WT (Fig. 1E,F,G). 2D class averages of all proteins are homogeneous (Fig. 1H,I,J).

**Fig. 2:**
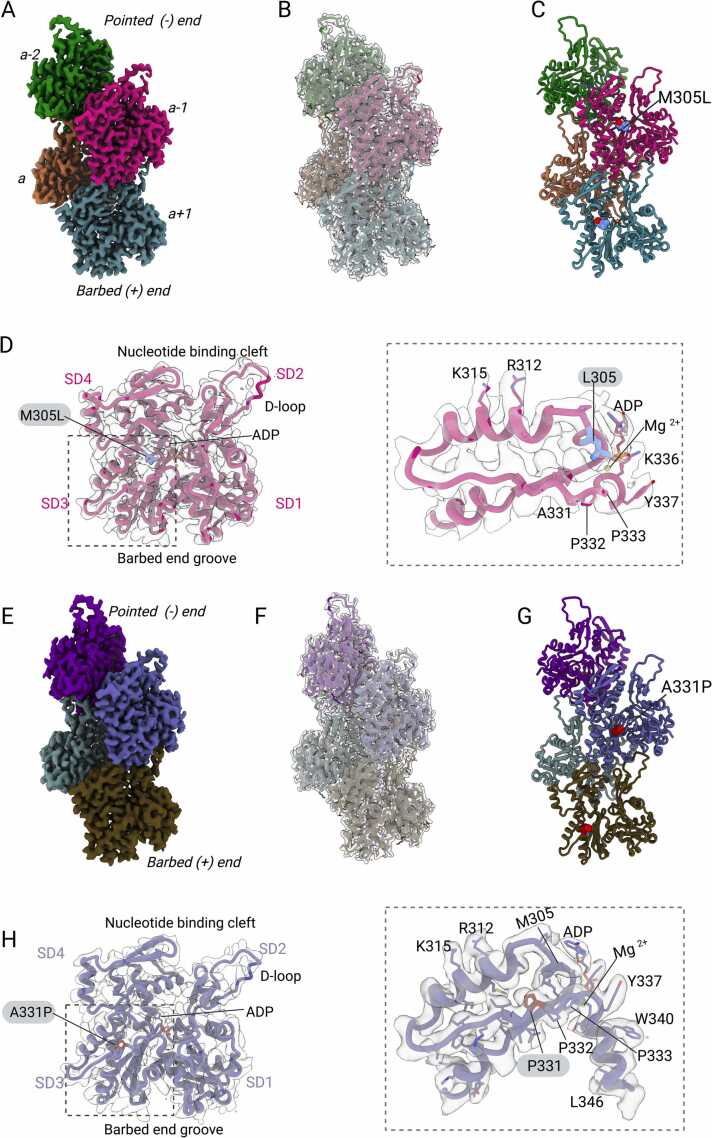
**Overall architecture of cardiac muscle α-actin mutants M305L and A331P. (A)** Cryo-EM reconstruction of M305L. Four protomers, indicated as *a*, *a+1*, *a-1*, and *a-2,* are shown. The barbed (+) end and the pointed (-) end are indicated. **(B)** Model of M305L (cartoon representation) fitted in the electron density map (transparent surface representation). Individual protomers are color coded according to (A). **(C)** Model of M305L in cartoon representation. The mutated residue L305 is shown in sphere representation. The bound ADP and Mg^2+^ are shown in stick and sphere representation, respectively. Individual protomers are color coded according to (A). **(D)** Position of M305L (blue) in the actin protomer (cartoon representation) with respect to the nucleotide binding cleft, the D-loop, the barbed end groove, and the bound ADP (stick representation) and Mg^2+^ (sphere). Actin subdomains are indicated. Cryo-EM densities are shown in transparent surface representation. **(E)** Cryo-EM reconstruction of A331P. Four protomers are shown. **(F)** Model of A331P (cartoon representation) fitted in the electron density map (transparent surface representation). Individual protomers are color coded according to (E). **(G)** Model of A331P in cartoon representation. The mutated residue P331 is shown in sphere representation. The bound ADP and Mg^2+^ are shown in stick and sphere representation, respectively. Individual protomers are color coded according to (E). **(H)** Position of A331P (red) in the actin protomer (cartoon representation) with respect to the nucleotide binding cleft, the D-loop, the barbed end groove, and the bound ADP (stick representation) and Mg^2+^ (sphere). Actin subdomains are indicated. Cryo-EM densities are shown in transparent surface representation.

We used our established cryo-EM processing strategy to solve the structure of filamentous M305L in the Mg^2+^⋅ADP state to a global resolution of 3.12 Å (Fig. 2A,B,C. Fig. S1A) (Arora et al., 2023). The structure of A331P in the Mg^2+^⋅ADP state was solved to a global resolution of 3.42 Å (Fig. 2E-G, Fig. S1C). The cryo-EM maps show local resolutions ranging from 1.5 Å to 4.0 Å (Fig. S1B,D). High local resolutions are present within the central core of the actin filament. Lower local resolutions are present at the filament surface and within surface loops (Fig. S1B,D). The quality of the maps allowed us to build unambiguous models for both mutant structures which feature resolved secondary structure elements, information for main chain and side chain orientations, and clear densities for Mg^2+^ and ADP (Fig. S1E). Further, the posttranslational modification of the key residues including the methylated H73 (H73^me^) are apparent in our cryo-EM reconstructions (Fig. S1E,F).

### 3.2 Overall architecture of cardiac muscle α-actin mutants M305L and A331P

Filamentous cardiac muscle α-actin mutants M305L and A331P share the well-known structural features of a two-stranded helix formed by head-to-tail interactions between individual actin protomers in each strand (Fig. 2A-C, E-G) (Ali et al., 2022, Arora et al., 2023, Chou and Pollard, 2019, Dominguez and Holmes, 2011, Egelman et al., 1982, Fujii et al., 2010, Galkin et al., 2015, Holmes et al., 1990, Mei et al., 2020, Oda et al., 2009). The protomer folds in four subdomains (SD) referred to as SD1 to SD4 (Fig. 2D) (Kabsch et al., 1990). The active site is in the nucleotide binding cleft between SD2 and SD4 in the center of the protomer (Fig. 2D) (Dominguez and Holmes, 2011, Pollard, 2016). ADP and Mg^2+^ are bound to the active sites of our structures (Fig. S1E, Fig. 2). The major binding interface with myosin motors and tropomyosin is the barbed end groove located between SD1 and SD3 (Dominguez and Holmes, 2011, Pollard, 2016, von der Ecken et al., 2016, von der Ecken et al., 2015). Mapping of HCM mutations M305L and A331P onto the three-dimensional structure of the actin protomer shows that both are located in SD3 (Fig. 2D,H). Mutation M305L is located ∼ 45 Å towards the pointed (-) end of the actin filament relative to A331P in the actin protomer (Fig. 2D,H). The observed helical rise of ∼28.7 Å and twist of −166.3° of both structures agrees with previously solved structures of native cardiac muscle α-actin (PDB ID: 8DMY) (Arora et al., 2023). The Cα atoms of structures M305L and A331P superimpose with a root mean square deviation (RMSD) of 0.15 Å and 0.468 Å to the structure of native cardiac muscle α-actin, suggesting that neither missense mutation drastically changes the overall architecture of the protein.

### 3.3 Interprotomer interactions in cardiac muscle α-actin mutants M305L and A331P

**Fig. 3:**
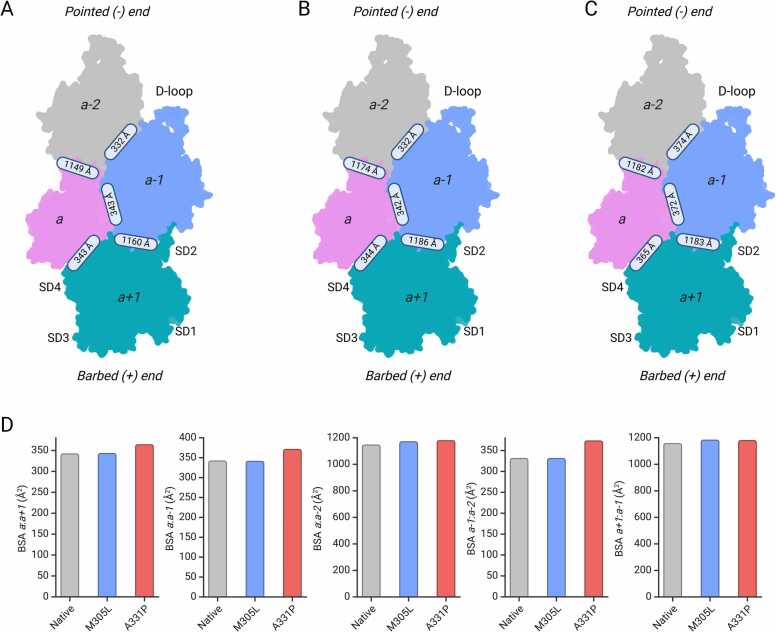
**Interprotomer interactions in HCM mutants M305L and A331P. (A)** Interprotomer contacts in native cardiac muscle α-actin (PDB ID: 8DMY). **(B)** Interprotomer contacts in M305L. **(C)** Interprotomer contacts in A331P. Note that in (A)-(C) labels represent the approximate location of the interprotomer interface in the actin filament. Individual protomers are color coded. **(D)** Plot of the BSA between neighboring actin protomers in native cardiac muscle α-actin, M305L, and A331P. Significant changes in BSA were only observed along the short pitch helix in M305L and both the long pitch helix and the short pitch helix in A331P compared to native cardiac muscle α-actin.

Analysis of the buried surface areas (BSA) between individual protomers in the actin filament shows differences in A331P and M305L compared to native cardiac muscle α-actin (Fig. 3). Bigger differences in BSA were observed along the short pitch helix in both mutant structures. Differences in the BSA along the long pitch helix were only observed in A331P. Specifically, the BSA between protomers *a* and *a-2* is increased by ∼25 Å^2^ while the BSA between protomers *a-1* and *a+1* is increased by ∼26 Å^2^ in M305L compared to native cardiac muscle α-actin (Fig. 3B,D). The BSA between protomers *a* and *a-1* in A331P is increased by ∼30 Å^2^, while the BSA between *a-1* and *a-2* is increased by ∼40 Å^2^. The BSA between protomers *a-1* and *a+1* in A331P is increased by ∼23 Å^2^ compared to native cardiac muscle α-actin (Fig. 3C,D). Comparative analysis suggests that mutations M305L and A331P increase the total BSA by ∼ 51 Å^2^ and ∼ 93 Å^2^, respectively. We also analyzed the solvation free energy (ΔG) of interprotomer interfaces using the Protein Interfaces, Surfaces and Assemblies (PISA) program (Shrake and Rupley, 1973). The short-pitch helices formed by (*a+1*) and (*a-1*), as well as the (*a*) and (*a-2*) protomer interfaces in A331P, show a favorable ΔG of ∼ −19.4 kcal/mol compared to WT and M305L actin filaments (ΔG of ∼ −18.7 kcal/mol). This shows that the interprotomer interfaces in A331P are more stable than those of WT and M305L. The BSA controls interprotomer contacts and the intrinsic protein stability (Marsh, 2013). The larger the BSA is the more rigid the filament is and vice versa. The overall increase in BSA in A331P and M305L suggests an overall increased rigidity compared to native cardiac muscle α-actin. This effect is more pronounced in A331P than in M305L. In addition, the normal mode analysis (NMA) of WT, M305L, and A331P actin filaments shows that WT actin is more flexible compared to M305L and A331P filaments (Fig. S2).

### 3.4 Structural features of cardiac muscle α-actin mutant M305L

**Fig. 4:**
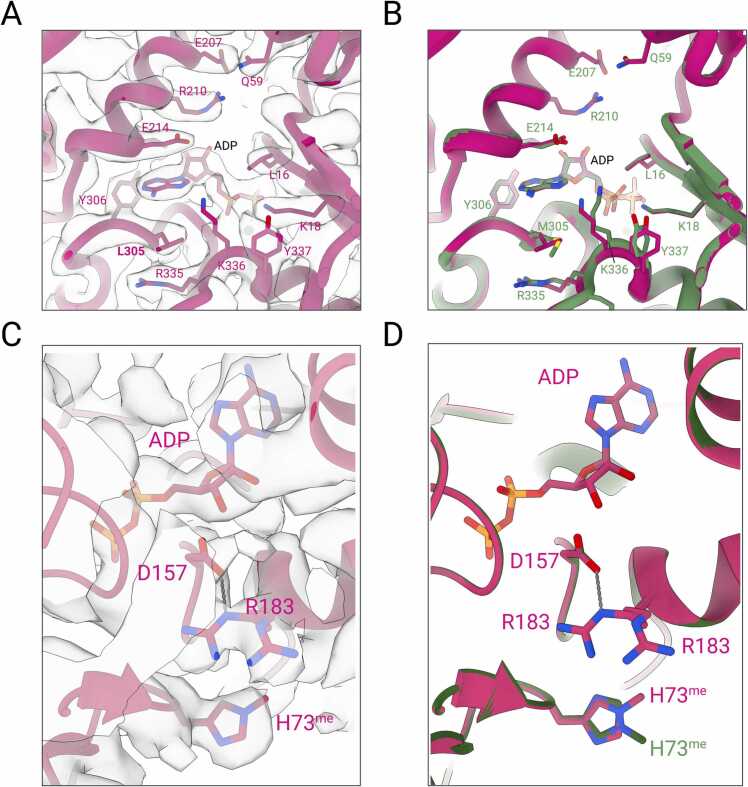
**Structural features of cardiac muscle α-actin mutant M305L. (A)** Structural alterations in the nucleotide binding cleft active site in M305L (cartoon representation). Cryo-EM densities are shown in transparent surface representation. **(B)** Superimposition of the nucleotide binding cleft active sites of M305L (pink) and native cardiac muscle α-actin (PDB ID: 8DMY, green). **(C)** Residue H73^me^ in M305L (pink cartoon) fitted in the cryo-EM densities (transparent surface representation). **(D)** Superimposition of M305L (pink) and native cardiac muscle α-actin (PDB ID: 8DMY, green) shows the altered side chain position of H73^me^.

M305 is located at the top of the nucleotide binding cleft active site where it supports the positioning of the adenine ring of the bound nucleotide. Further, M305 supports the positioning of the bound ADP and Mg^2+^ in the nucleotide binding cleft active site through hydrophobic interactions in the structure of the native cardiac muscle α-actin (Arora et al., 2023). The substitution of the *S*-methyl thioether side chain of methionine at position 305 with the isobutyl side chain of leucine causes distant changes in the positioning of side chains of residues L16, Q59, E214, M305, R335, K336, Y337, and the conformation of the phosphate groups of the ADP (Fig. 4). The most prominent changes are seen in the side chains of residues R335-Y337 located in a loop that connects the outer domain formed by SD1 and SD2 and the inner domain formed by SD3 and SD4 of the actin protomer. In addition, the side chain of residue R183 in the active site adopts alternate conformations, as does the proximal H73^me^ (Fig. 4A-D). The analysis of the ADP binding site near residue L305 in the nucleotide binding cleft reveals an accessible surface area (ASA) of 68.28 Å^2^, a BSA of 20.73 Å^2^, and a solvation free energy effect (ΔG) of 0.11 kcal/mol. By contrast, the structural analysis of the respective site in native cardiac muscle α-actin reveals an ASA of 75.10 Å^2^, a BSA of 26.31 Å^2^, and a ΔG of 0.29 kcal/mol.

### 3.5 Structural features of cardiac muscle α-actin mutant A331P

**Fig. 5:**
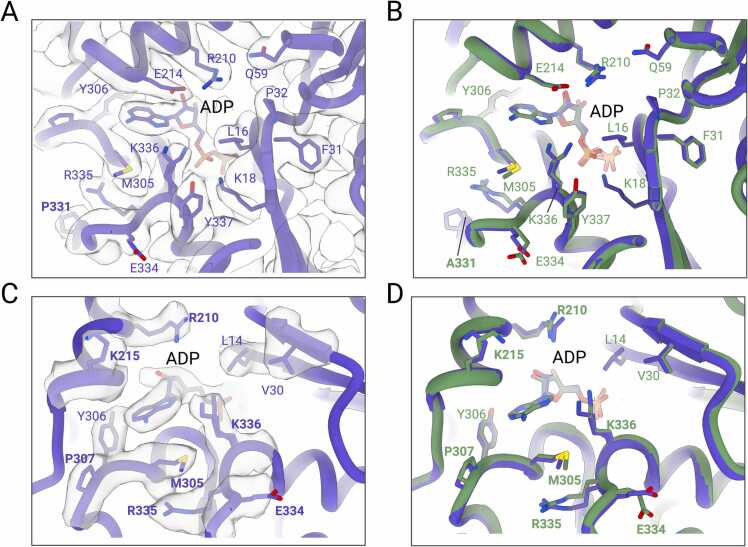
**Insights into the nucleotide binding cleft active site of A331P.** (**A**) Structural alterations in the nucleotide binding cleft active site in A331P (cartoon representation). Cryo-EM densities are shown in transparent surface representation. Alternate conformations of the rotamers of K336 are shown. Residue A331P is indicated with a boldface label. (**B**) Superimposition of the nucleotide binding cleft active sites of A331P (blue) and native cardiac muscle α-actin (PDB ID: 8DMY, green). (**C**) Close-up view of the nucleotide binding cleft active site in A331P (blue) with cryo-EM densities shown in transparent surface representation. (**D**) Superimposition of the nucleotide binding cleft active sites of A331P (blue) and native cardiac muscle α-actin (PDB ID: 8DMY, green). Altered residue conformations in the nucleotide binding cleft active sites are indicated with boldface labels.

The substitution of an alanine at position 331 with a cyclic pyrrolidine side chain of proline creates a sequence of three consecutive prolines (Fig. 1B) within SD3 of the actin protomer with implications for function. The absence of stacking interactions between the three polyproline pyrrolidine rings suggests increased local flexibility. A331P is located ∼15.8 Å from the α-PO_4_ group of the bound ADP in the nucleotide binding cleft active site. Further, analysis of the residue A331 in native cardiac muscle α-actin reveals an ASA of 38.94 Å^2^, compared to an ASA of 57.40 Å^2^ in HCM mutant P331. The presence of a proline at position 331 results in local changes in sidechain positions of active site residues compared to the native protein. Specifically, side chain orientations of residues E334, R335, and K336 in the hinge at the base of the ADP and M305, E214, and R210 at the top of the active site differ from those of the native protein (Fig. 5). The position of ADP in the A331P active site differs by ∼ 0.7 Å compared to the structure of native cardiac muscle α-actin. Notably, the side chain of K336 in the hinge region at the base of ADP adopts two alternate conformations, as shown in the electron density maps (Fig. 5). One conformation allows for interaction with the adenine ring of ADP, while the other conformation allows interaction with the oxygen from the α-PO_4_ group of the bound ADP. These changes result in an ASA of 99.12 Å^2^ and a BSA of 32.2 Å^2^ for K336 at the base of ADP compared to an ASA of 83.87 Å^2^ and a BSA of 28.86 Å^2^ for K336 in the native protein. These rearrangements of K336 in the A331P mutant induce a ΔG of 0.24 kcal/mol compared to −0.02 kcal/mol of K336 in native cardiac muscle α-actin. In addition, residue R210 at the top of ADP in the nucleotide binding cleft active site adopts a different conformation with an ASA of 87.70 Å^2^ and a BSA of 13.69 Å^2^ compared to an ASA of 68.96 Å^2^ and BSA of 10.39 Å^2^ for R210 in native cardiac muscle α-actin.

### 3.6 Structural implications of mutations M305L and A331P on the interaction with tropomyosin

**Fig. 6:**
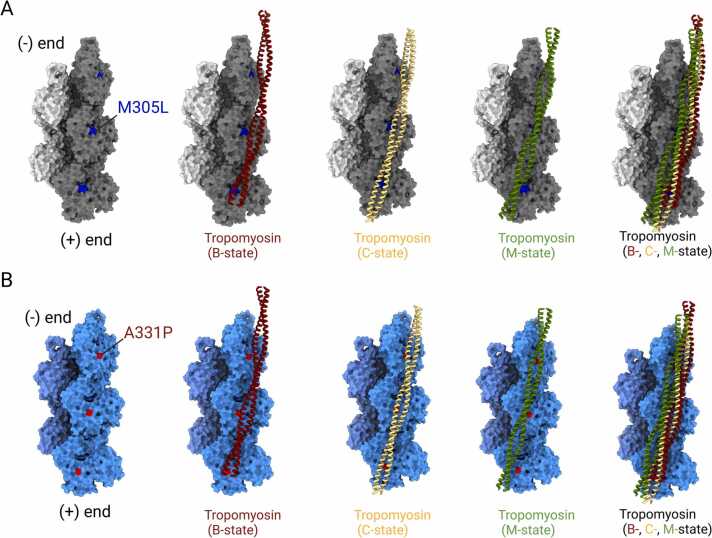
**Interactions between M305L and A331P with tropomyosin in different functional states. (A)** Superimposition of the structure of M305L with tropomyosin in the B-, C-, and M-states (PDB IDs: 5JLF, 5NOG, 5JLH; cartoon representation). Five M305L protomers are shown in surface representation. The mutated residue is shown as blue sphere. **(B)** Superimposition of the structure of A331P with tropomyosin in the B-, C-, and M-states (PDB IDs: 5JLF, 5NOG, 5JLH; cartoon representation). Five A331P protomers are shown in surface representation. The mutated residue is shown as red sphere.

Tropomyosin is an elongated α-helical dimer that binds to seven actin protomers in the actin filament in different azimuthal positions that correspond to different functional states (Li et al., 2011, McKillop and Geeves, 1993, von der Ecken et al., 2015). The interaction between both actin and tropomyosin is driven by weak electrostatic interactions (Li et al., 2011, von der Ecken et al., 2016, von der Ecken et al., 2015). Comparison of the M305L and A331P mutants with the published structures of tropomyosin in the functional B-, C-, and M-states (PDB IDs: 5JLF, 5NOG, and 5JLH) reveals discrete interaction profiles with various degrees of structural superposition at the tropomyosin binding site (Fig. 6). This analysis further shows that the M305L residue is positioned farthest from tropomyosin in the B-state. Conversely, the M305L residue is in closer proximity to tropomyosin in the M-state (Fig. 6A). In contrast, structural superimposition reveals that the A331P residue is positioned farthest from tropomyosin in the B-state and closest to tropomyosin in the C-state, respectively (Fig. 6B). The structural overlay of tropomyosin with the A331P mutant shows more occupancy over five subunits of filamentous actin compared to the occupancy of tropomyosin with the M305L mutant, suggesting a more pronounced effect of the mutation on the interaction with tropomyosin.

### 3.7 Structural implications of mutations M305L and A331P on the interaction with myosin

Force production and muscle contraction require the regulated interaction between F-actin and myosin motors (Gordon et al., 2000). The binding of myosin on actin filaments displaces tropomyosin from the C-state to the open M-state (Gordon et al., 2000, Holmes and Lehman, 2008). The well-characterized binding interface between both proteins encompasses the barbed end groove of the actin filament and several structural elements in the myosin motor domain. These include, but are not limited to loop-2, loop-4, and the cardiomyopathy (CM)-loop (Robert-Paganin et al., 2020, von der Ecken et al., 2016). The latter has been previously identified as a hotspot for HCM mutations in the myosin motor domain (Dausse et al., 1993, Geisterfer-Lowrance et al., 1990).

**Fig. 7:**
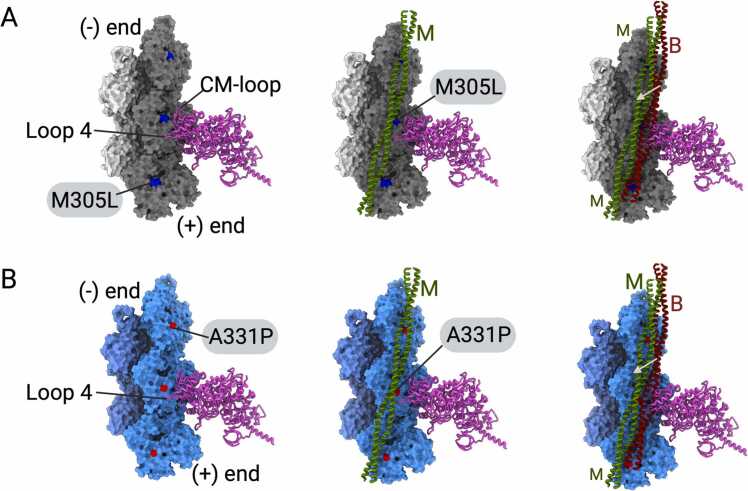
**Interactions between M305L and A331P with the cardiac myosin motor domain in the rigor state. (A)** Superimposition of the structure of M305L with myosin in the rigor state (pink cartoon) (PDB ID: 5JLH). Five M305L protomers are shown in surface representation. The mutated residue is shown as blue sphere. **(B)** Superimposition of the structure of A331P with myosin in the rigor state (pink cartoon) (PDB ID: 5JLH). Five A331P protomers are shown in surface representation. The mutated residue is shown as red sphere. The white arrow indicates the movement of tropomyosin from the B-state to the M-state.

Superimposition of the published structures of the nonmuscle myosin-2C motor domain and the cardiac myosin-2 motor domain bound to the thin filament (PDB IDs: 5JLH, 7JH7, 8EFH) with M305L revealed proximal interactions with the myosin CM-loop located between the active site of M305L and the actin-binding region in the motor domain (Fig. 7A, Fig. S3,4). Specifically, the ADP molecule in the M305L active site is within the van der Waal’s distance from the CM-loop of myosin in the rigor state (Fig. S4). The hydrophobic L305 residue shields ADP on one side and distinct subtle changes in the active site could affect myosin binding. Whereas A331P forms van der Waal interactions with the actin-binding loop-4 of the myosin motor domain (Fig. 7B, Fig. S4). However, the three prolines A331P, P332, and P333 residues position in between loop-4 and CM-loop. The subtle conformation changes induced by the A331P mutation could affect myosin binding more than the M305L mutation as multiple regions involved in myosin binding are affected in the A331P mutant.

## 4 Discussion

HCM is a debilitating disease that affects around 1 in 500 individuals (McKenna and Judge, 2021). While the clinical aspects of HCM are well understood, the molecular mechanisms that trigger the initiation of cardiac remodeling pathways that lead to HCM remain largely elusive (Van Driest et al., 2003). 12 clinically relevant missense mutations in *ACTC1* have been reported to cause HCM (Despond and Dawson, 2018, Kaski et al., 2009, Mogensen et al., 1999, Mogensen et al., 2004, Morita et al., 2008, Olivotto et al., 2008, Olson et al., 2000, Van Driest et al., 2003). Mutations M305L and A331P are located on the surface of the actin filament and induce structural alterations in interprotomer interfaces and the nucleotide binding cleft active site without changing the conserved structural characteristics of native cardiac muscle α-actin nor the ability of the mutant protein to form filaments (Fig. 1, Fig. 2, Fig. 3, Fig. 4, Fig. 5, Fig. 6). Notably, our structural analysis shows that the BSA is increased in both mutants compared to the native protein (Fig. 3), resulting in more rigid filaments. A similar effect has been reported for phalloidin, which increases the rigidity of actin filaments 2-fold by strengthening interprotomer interactions and alters the interaction between filamentous actin and actin-binding proteins (Diensthuber et al., 2011, Isambert et al., 1995, Pospich et al., 2020). For instance, the strengthening of protomer-protomer contacts through the addition of phalloidin to activated skinned cardiac muscle fibers causes an increase in the active force (Bukatina et al., 1995). Thus, it is plausible that the increased rigidity of mutant actin filaments affects force generation in the sarcomere, which may lead to altered cardiac function and contribute to the development of HCM.

In addition, it was previously reported that the melting temperature of A331P is slightly higher than that of the native protein (Mundia et al., 2012), suggesting increased stability. Given the essential role of flexural rigidity for proper function, it is plausible that the reduced flexibility in M305L and A331P contributes to altered interactions with muscle regulatory proteins (Isambert et al., 1995). This is further supported by the observation that both HCM mutations are in the barbed end groove, suggesting that altered interactions with myosin, tropomyosin, and potentially other sarcomeric actin binding proteins contribute to the molecular mechanism underlying HCM.

Our structural data of M305L together with published biochemical work support a model in which interactions with myosin and tropomyosin are altered. While the existing literature on the molecular mechanisms of M305L is inconsistent, it was shown that the extent to which M305L activates the myosin ATPase activity is reduced by ∼ 50 % in the absence and presence of tropomyosin, without changing the apparent affinity for F-actin under steady-state conditions (Muller et al., 2012). This agrees with the observation that the superimposition of M305L and the myosin motor domain in the rigor state does not perturb the actin-myosin interaction. Further, mutations in the actin:CM-loop interface have previously been shown to reduce the actin-activated ATPase activity of myosin and to increase the ensemble force of β-cardiac myosin that lead to hypercontractility at the molecular scale and HCM in the heart (Sommese et al., 2013). While the precise effect of mutation M305L in cardiac muscle α-actin on myosin motor function cannot be predicted, it is possible that the mutation phenocopies the effect reported for the actin:CM-loop interface and thus contributes to disease pathogenesis. In addition, The observation that the landmark residue C374 is buried in M305L rather than being surface exposed also suggests that altered post-translational modification patterns such as S-glutathionylation or S-nitrosylation may be altered and contribute to disease pathogenesis (Dalle-Donne et al., 2003, Garcia-Ortiz et al., 2017).

Superimposition of published tropomyosin structures on the cryo-EM structure of M305L showed that the highest overlap is present between M305L and tropomyosin in the functional M-state while the lowest degree of overlap between both proteins is present in the B-state. This agrees with a recent study that reported that *Drosophila* M305L increases contractile activation and prolongs inactivation through the inhibition of the movement of tropomyosin from the M-state to the B-state (Viswanathan et al., 2020).

The unchanged overall architecture and filament characteristics of M305L suggest that the mutant actin can copolymerize with native cardiac muscle α-actin in heterozygous HCM patients. This finding aligns with the ‘poisonous peptide’ hypothesis and indicates a dominant-negative effect (Erdmann et al., 2021, Schwartz, 1995). Indeed, previous biochemical and *in vivo* studies showed the copolymerization of native and mutant cardiac muscle α-actin into filaments (Muller et al., 2012, Viswanathan et al., 2020). It has also been shown that increasing ratios of M305L in mixtures with native cardiac muscle α-actin reduce the actin-activation of the myosin ATPase activity (Muller et al., 2012). Based on the analysis of cryo-EM micrographs, we did not notice a change in the filament appearance as previously reported (Muller et al., 2012). We attribute this difference to subtle biochemical differences in posttranslational modification patterns of recombinant actin produced in insect cells compared to the actin produced in mammalian cells in this study.

Interestingly, the observation that the side chain conformation of R183 and H73me in the active site adopt different conformations (Fig. 4A-D) may contribute to the reported increased P_i_ release rate of M305L compared to the native protein (Mundia et al., 2012). While previous structural and biochemical studies suggest that R183 is not part of the primary P_i_ egress pathway, disruption of the putative R183 backdoor increases the rate of P_i_ release to a similar extent as reports for M305L (Oosterheert et al., 2023).

Our cryo-EM structure of A331P together with molecular modeling of the interactions with myosin and tropomyosin suggests subtle alterations in the interaction mechanisms between the proteins. The observation that the overall architecture of A331P is not changed compared to cardiac muscle α-actin agrees with previous biochemical and cell biological studies on mammalian cardiac muscle α-actin A331P but contrasts published work on yeast A331P (Mundia et al., 2012, Vang et al., 2005, Wong et al., 2001). The observed side chain polymorphism of K336 in the A331P structure suggests increased local flexibility within the hinge region of the protomer, which is critical for the protein during filament formation (Dominguez, 2019). It is plausible that the side chain polymorphism contributes to the altered interprotomer contacts in A331P filament protomers compared to the native protein. Notably, mutation of K336 in skeletal muscle α-actin causes congenital myopathy by reducing the affinity of myosin and actin-binding proteins by altering interactions between actin protomers but without changing the overall architecture of the filament (Umeki et al., 2019). Likewise, mutation of hinge region residue E334 in Baraitser-Winter cerebrontofacial syndrome reduces the affinity of myosin for γ-actin under rigor conditions (Greve et al., 2024a).

The superimposition of published structures of myosin with our structure of filamentous A331P shows interactions between loop-4 of the myosin motor domain and the mutated residue. Loop-4 has previously been shown to contribute to the stabilization of the actin-myosin interface, particularly in the weak actin-binding states (Gyimesi et al., 2008). While we cannot predict the consequences of this interaction on myosin function, the observation that A331P has the lowest and highest overlap with tropomyosin in the B-state and C-state, respectively, suggest implications for function during thin filament activation and relaxation. Indeed, recent docking and structural studies suggest a competition between contacts between myosin and tropomyosin and tropomyosin and actin (Doran et al., 2020, Doran et al., 2023). The superimposition of myosin and tropomyosin in different states suggests steric interference between tropomyosin in the B- and C-states with myosin that inhibits contraction. The small size of the proline side chain in A331P and the increased flexibility in the hinge region due to the presence of three adjacent prolines is likely to weaken the interaction with tropomyosin in general but with the highest effect in the C-state. This interpretation is supported by previous structural studies that showed that residue A331 is located in a loop that contains two lysine residues (K326, K328) that interact with negatively charged residues of tropomyosin in the C-state (Risi et al., 2017, Risi et al., 2021, von der Ecken et al., 2015). Further, it has been proposed that mutation A331P may introduce a kink in the loop which alters the positioning of K326 and K328 and thus the interaction with tropomyosin (Risi et al., 2021). Together, this suggests that in the presence of Ca^2+^, mutation A331P facilitates myosin binding which leads to the movement of myosin to the M-state and muscle contraction. The distance between the A331P residue and troponin is more than 15 Å on the thin filament, suggesting that the mutation is unlikely to directly influence the functional mechanisms of troponin (Risi et al., 2023).

This model is supported by published biochemical work on reconstituted thin A331P filaments that showed that myosin binding and cross-bridge kinetics are largely unchanged in the presence of Ca^2+^ (Bai et al., 2014). The reported slight decrease in the myosin duty ratio under Ca^2+^-free conditions may contribute to the previously observed decrease in relaxed tension in the reconstituted A331P thin filaments (Bai et al., 2014, Dahari and Dawson, 2015). Together, the observed subtle structural alterations in A331P filaments together with published biochemical data suggest that mutation A331P in cardiac muscle α-actin is likely to contribute to early cardiac remodeling pathways that result in HCM.

Comparative analysis of our high-resolution cryo-EM structures, together with published biochemical and functional work, suggest that both missense mutations alter the interactions between individual actin protomers in the filament, the interaction with tropomyosin, and the interaction with myosin in a complex and mutation-specific manner. It is also important to note that A331P has been shown to decrease the affinity of cardiac myosin binding protein-C, a key regulator of cardiac contractility, for actin (Chow et al., 2014). This underlines the complexity of the molecular mechanisms of HCM that lead to diverse phenotypic heterogeneity in HCM patients (Van Driest et al., 2003). Thus, it is conceivable that the missense mutations induce specific alterations in protein interaction networks within the muscle sarcomere. These changes may interfere with the native organization of thin and/or thick filaments and their highly regulated interaction, thereby triggering the initiation of cardiac remodeling pathways that lead to HCM.

Our structural investigations not only have implications for our understanding of actin structure and function in muscle but also for the regulation of cytoskeletal force generation and contractility in nonmuscle cells (Barua et al., 2014, Selvaraj et al., 2023). While nonmuscle cells lack troponin, tropomyosin assumes pivotal roles in the regulation of myosin motor activity (Gunning et al., 2008, Manstein et al., 2020). Homologous mutations to M305L and A331P in cardiac muscle α-actin have been reported in nonmuscle actin isoforms. Mutation M304T has been reported in cytoplasmic γ-actin and mutation A330V in cytoplasmic β-actin (Fig. 1B), suggesting potential parallels in their effects on the interaction with tropomyosin and myosin motors. In addition, residues T304 and Y306 located adjacent to M305L have been reported to be phosphorylated (Heissler and Chinthalapudi, 2024, Hornbeck et al., 2015). While the structural and functional consequences of these phosphorylation events are unknown, it is conceivable that the presence of a missense mutation at position 305 could alter the phosphorylation landscape of cardiac muscle α-actin, adding another level layer of complexity to the structural and molecular mechanisms associated with HCM.

## CRediT authorship contribution statement

**Krishna Chinthalapudi:** Writing – review & editing, Writing – original draft, Visualization, Validation, Supervision, Resources, Project administration, Methodology, Investigation, Funding acquisition, Formal analysis, Data curation, Conceptualization. **Sarah Heissler:** Writing – review & editing, Writing – original draft, Visualization, Validation, Supervision, Resources, Project administration, Methodology, Investigation, Formal analysis, Conceptualization. **Hsiang-Ling Huang:** Writing – review & editing, Methodology, Investigation, Formal analysis, Data curation. **Andrejus Suchenko:** Writing – review & editing, Visualization, Methodology, Investigation, Formal analysis, Data curation. **Giovanna Grandinetti:** Writing – review & editing, Methodology, Investigation, Formal analysis. **Mohan K. Balasubramanian:** Writing – review & editing, Supervision, Resources, Investigation, Funding acquisition, Conceptualization.

## Declaration of Competing Interest

The authors declare that they have no known competing financial interests or personal relationships that could have appeared to influence the work reported in this paper.
