# Supplementary material for: Cryo-EM structures of cardiac muscle α-actin mutants M305L and A331P give insights into the structural mechanisms of hypertrophic cardiomyopathy
Source: Eur J Cell Biol. 2024 Dec;103(4):151460. doi: 10.1016/j.ejcb.2024.151460 (PMC11611453; doi:10.1016/j.ejcb.2024.151460)
Supplement: Supplementary file 1 — Supplementary material [file mmc1.docx]

**Supplementary Information**

**
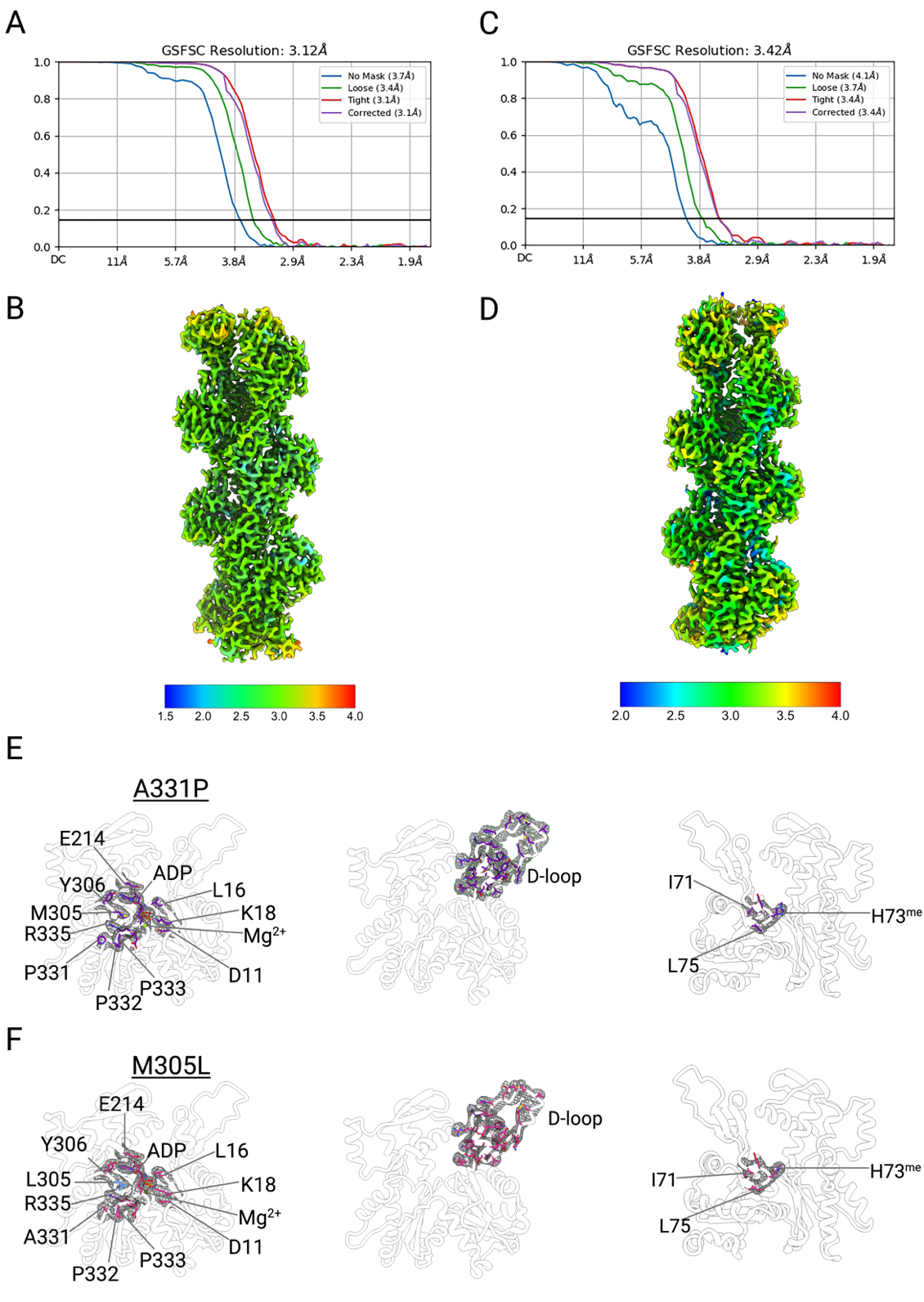
**

**Figure S1: Quality of the M305L and A331P cryo-EM maps and reconstructions. (A)** Gold-standard FSC curve. The resolution of M305L was determined by the FSC=0.143 criterion to 3.12 Å. **(B)** Local resolution of M305L. **(C)** Gold-standard FSC curve. The resolution of A331P was determined by the FSC=0.143 criterion to 3.42 Å. **(D)** Local resolution of A331P. **(E,F)** The nucleotide binding cleft active site (left), the D-loop (middle), and the methylated H73 (right) regions are highlighted with the respective electron density maps (grey mesh) for A331P and M305L (stick representation) models.


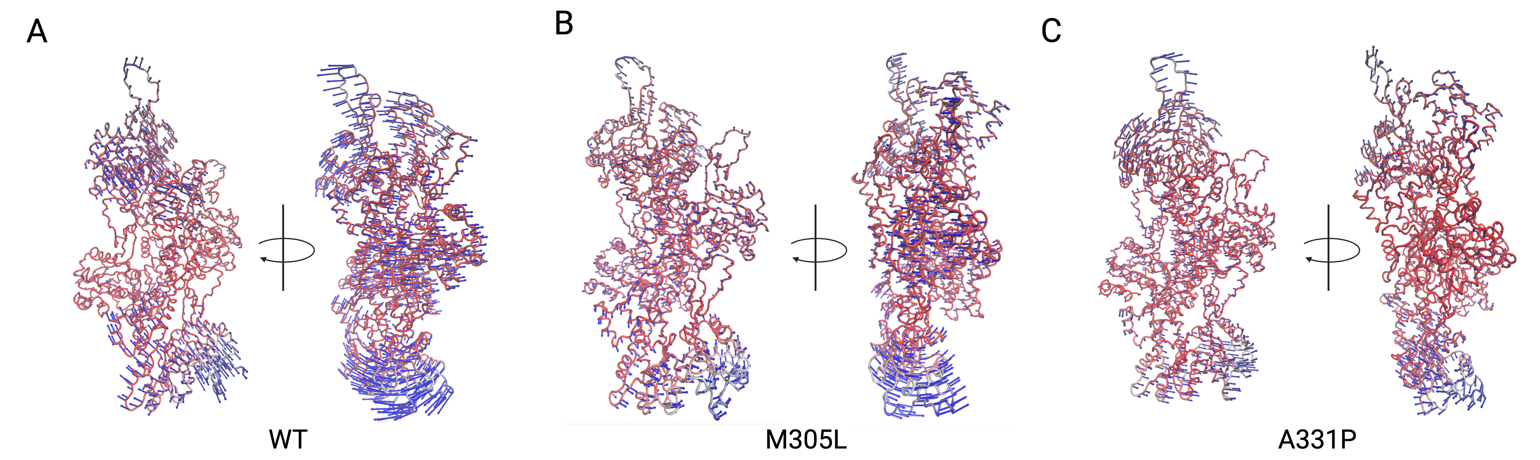


**Figure S2: Normal mode analysis of WT, A331P, and M305L actin filaments. (A)** Normal mode analysis of the WT (PDB: 8DMY) actin filament shows large-scale motions. **(B)** Normal mode analysis of the M305L mutant actin filament shows medium-scale motions compared to WT. **(C)** Normal mode analysis of the A331P mutant actin filament shows small-scale motions compared to WT. Eigenvectors are shown in blue color.


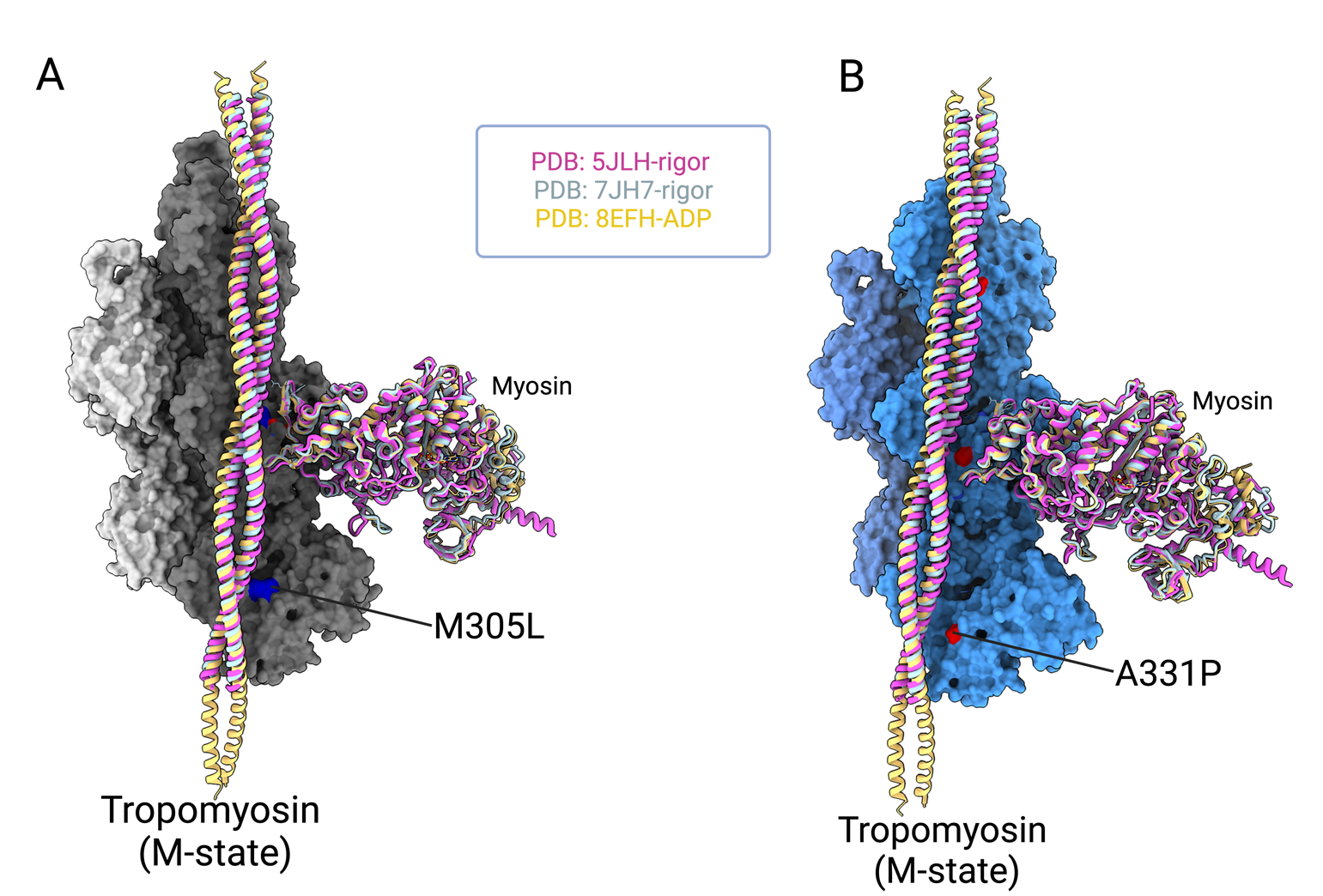


**Figure S3: Structural comparison of M305L and A331P with published actin-myosin-tropomyosin structures. (A)** Superimposition of M305L with published actin-myosin-tropomyosin structures (PDB ID: 5JLH, nonmuscle myosin-2C (rigor); PDB ID: 7JH7, β-cardiac myosin (rigor); PDB ID: 8EFH, β-cardiac myosin (ADP)). For clarity, only the mutant actin filament is shown. **(B)** Superimposition of A331P with published actin-myosin-tropomyosin structures (PDB ID: 5JLH, nonmuscle myosin-2C (rigor); PDB ID: 7JH7, β-cardiac myosin (rigor); PDB ID: 8EFH, β-cardiac myosin (ADP)). For clarity, only the mutant actin filament is shown.


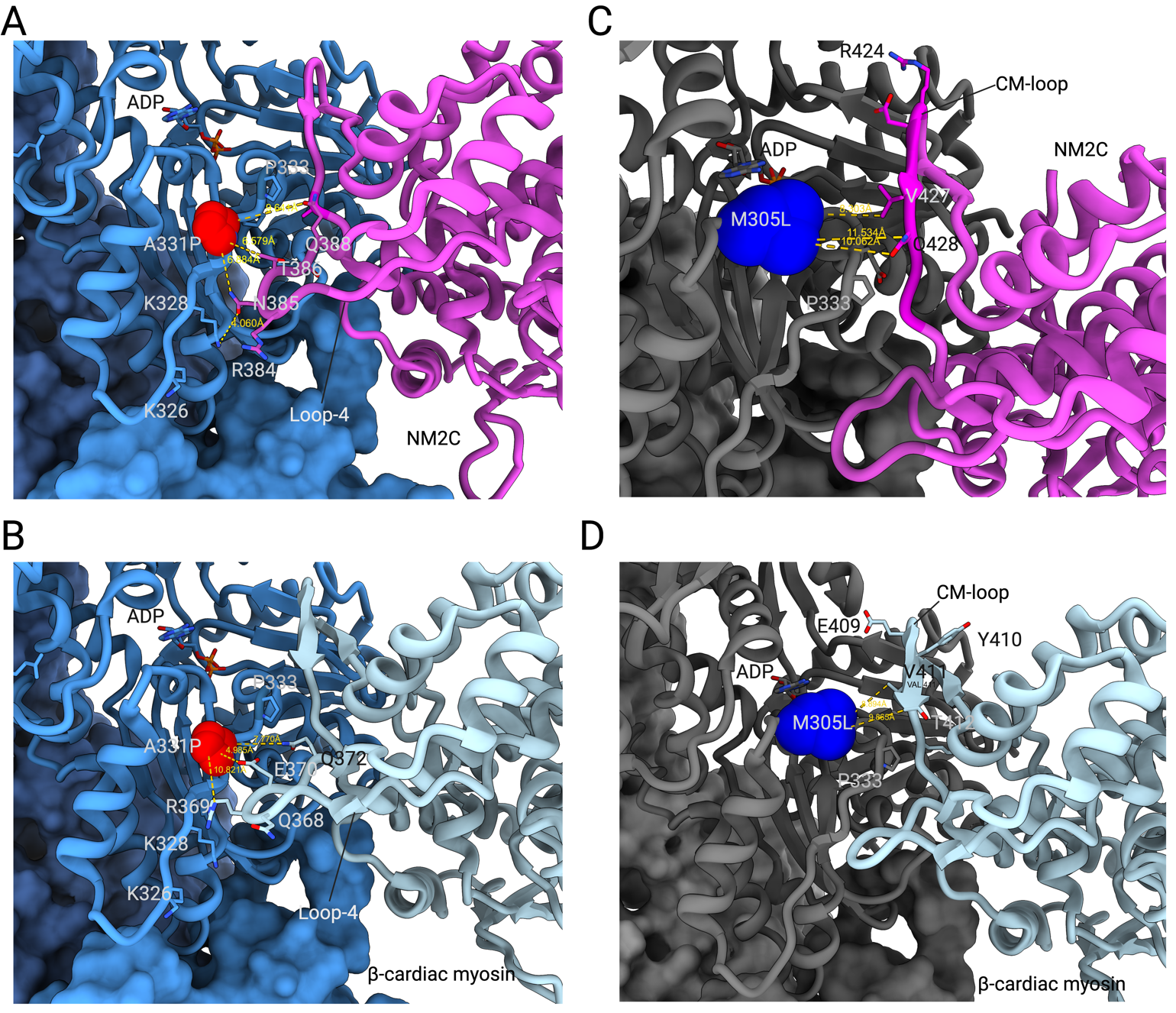


**Figure S4: Actin-myosin interface of A331P and M305L. (A)** Close-up view of the interface between the A331P filament (blue) and the nonmuscle myosin-2 motor domain (NM2C, pink) in the rigor state (PDB ID: 5JLH). Mutation A331P is shown as a red sphere. **(B)** Close-up view of the interface between the A331P filament (blue) and β-cardiac myosin (light blue) in the rigor state (PDB ID: 7JH7). Mutation A331P is shown as a red sphere. **(C)** Close-up view of the interface between the M305L filament (grey) and the nonmuscle myosin-2 motor domain (pink) in the rigor state (PDB ID: 5JLH). Mutation M305L is shown as a blue sphere. **(D)** Close-up view of the interface between the M305L filament (grey) and β-cardiac myosin (light blue) in the rigor state (PDB ID: 7JH7). Mutation M305L is shown as a blue sphere.
